# Sustainable Soil Management: Effects of Clinoptilolite and Organic Compost Soil Application on Eco-Physiology, Quercitin, and Hydroxylated, Methoxylated Anthocyanins on *Vitis vinifera*

**DOI:** 10.3390/plants12040708

**Published:** 2023-02-05

**Authors:** Eleonora Cataldo, Maddalena Fucile, Davide Manzi, Cosimo Maria Masini, Serena Doni, Giovan Battista Mattii

**Affiliations:** 1 DAGRI, Department of Agriculture, Food, Environment, and Forestry Sciences and Technologies, University of Florence, 50019 Sesto Fiorentino, FI, Italy; 2 DN360 Piazza d’Ancona, 3, 56127 Pisa, PI, Italy; 3 CNR IRET, Via Moruzzi, 1, 56124 Pisa, PI, Italy

**Keywords:** Zeowine, gas exchanges, grapevine, water stress, composting process, soil management

## Abstract

Climate change and compostinS1g methods have an important junction on the phenological and ripening grapevine phases. Moreover, the optimization of these composting methods in closed-loop corporate chains can skillfully address the waste problem (pomace, stalks, and pruning residues) in viticultural areas. Owing to the ongoing global warming, in many wine-growing regions, there has been unbalanced ripening, with tricky harvests. Excessive temperatures in fact impoverish the anthocyanin amount of the must while the serious water deficits do not allow a correct development of the berry, stopping its growth processes. This experiment was created to improve the soil management and the quality of the grapes, through the application of a new land conditioner (Zeowine) to the soil, derived from the compost processes of industrial wine, waste, and zeolite. Three treatments on a Sangiovese vineyard were conducted: Zeowine (ZW) (30 tons per ha), Zeolite (Z) (10 tons per ha), and Compost (C) (20 tons per ha). During the two seasons (2021–2022), measurements were made of single-leaf gas exchange and leaf midday water potential, as well as chlorophyll fluorescence. In addition, the parameters of plant yield, yeast assimilable nitrogen, technological maturity, fractionation of anthocyanins (Cyanidin-3-glucoside, Delphinidin-3-glucoside, Malvidin-3-acetylglucoside, Malvidin-3-cumarylglucoside, Malvidin-3-glucoside, Peonidin-3-acetylglucoside, Peonidin-3-cumarylglucoside, Peonidin-3-glucoside, and Petunidin-3-glucoside), Caffeic Acid, Coumaric Acid, Gallic Acid, Ferulic Acid, Kaempferol-3-O-glucoside, Quercetin-3-O-rutinoside, Quercetin-3-O-glucoside, Quercetin-3-O-galactoside, and Quercetin-3-O-glucuronide were analyzed. The Zeowine and zeolite showed less negative water potential, higher photosynthesis, and lower leaf temperature. Furthermore, they showed higher levels of anthocyanin accumulation and a lower level of quercetin. Finally, the interaction of the beneficial results of Zeowine (soil and grapevines) was evidenced by the embellishment of the nutritional and water efficiency, the minimizing of the need for fertilizers, the closure of the production cycle of waste material from the supply chain, and the improvement of the quality of the wines.

## 1. Introduction

Climate change and the problem of corporate sustainability (organic and closed-loop companies) are two highly topical and relevant issues of the twenty-first century [1,2].

The optimization of the composting methods in closed-loop corporate chains can skillfully address the waste problem (pomace, stalks, and pruning residues) in viticultural areas [3,4]. The wine industry produces enormous quantities of waste every year along the production chain [5,6]. In general, the grape marc represents 20–30% of the weight of the grapes used to make wine [7]. It is estimated that about 5% of the total volume of wine produced in Tuscany (an Italian wine region) in a year constitutes wine lees residues (i.e., more than 100,000 hl) [8]. Briefly, one elaborated ton of berries roughly engenders 1.65 m^3^ of wastewater, 0.13 tons of marc, 0.06 tons of lees, and 0.03 tons of stalks [9]. With a view to sustainability and a zero-waste circular economy, in recent years new strategies have been developed to enhance the blooming of the marketable products obtained from industrial waste recovery operations [10,11,12,13,14]. However, nowadays most wine entrepreneurs claim to deliver the pomace to the distillery for the production of Grappa, while they dispose of the waste stalks, lees, and wastewater [15,16,17]. As foreseen in ISO_14000 and ISO_14001, the wine trade is required to downsize its ambient impact, by adopting environmentally friendly technologies and strategies, which allow, for instance, the lowering of water consumption, the recycling of by-products, and the lowering of waste [18,19].

Fortunately, the scientific community has recently invested an increasing amount of energy into projects sensitive to this issue. The bio-compost obtained by sheep manure (*Ovis aries* L.), grapevine marc (*Vitis vinifera* L.), and mango (*Mangifera indica* L.) leaves was rated in its performance (i.e., microbiological, physical–chemical, and nutritional parameters) for agriculture use [12]. In Stellenbosch, South Africa, wine-filter and pruning wastes added to berry skins and seeds were successfully composted (21.25 C (%), 1.86 N (N%), 0.86 Ca (%), 31.15 B (%), and 52.59 Mn (%)) [14]. A good quality compost (NH_4_^+^-N/NO_3_—N < 0.5, low levels of heavy metals, 0.2–0.6 dS m^−1^, and high germination index) was produced with winery wastewater sludge + grape stalks [20]. In Serra Gaucha, the impact in terms of heavy metals (copper, zinc, and chromium) was assessed during the composting process of the grape industrialization by-products; the study established that the product represented a skilled raw material that could be employed for certifiable organic agriculture: satisfactory ranks of organic matter and adequate essential components [21]. On the one hand, grape marc vermicompost application counteracted the low pH of the grape marc and attenuated the high phytotoxicity and polyphenol content, and on the other, it increased the α- β- variegation of the bacterial population at the taxonomic/phylogenetic levels [22].

Intimately interconnected with the problem of residue management is that of the preserving of the expression of the European wine heritage in the scenario of the delicate issue of global warming by coping with the alterations and imbalances originating from the unpredictable climatic conditions [23]. Temperature and water balance are the uppermost drivers of vine growing and regulate the flowering, the pre-closure of the bunch, veraison, and harvest [24,25]. Owing to the ongoing global warming, in many wine-growing regions, there has been unbalance in the ripening, with tricky harvests (i.e., in Bordeaux, Spain, Italy, and India) [26,27,28,29,30]. In fact, excessive temperatures impoverish the acidic content of the must [31] and stimulate the gathering of (PAL) phenylalanine ammonia-lyase mRNA [32] (an environmental stress marker [33]), while the serious water deficits do not allow a correct development of the berry, stopping its growth processes. In fact, it was demonstrated that water stress (deficit threshold, more than 50% of ETc-evapotranspiration during the bud break and bloom step) can reduce the yield [34]. Furthermore, water scarcity, depending on the level and period, can also affect the technological composition of the berries [35,36,37]. From the point of view of sugars, two distinct scenarios open up: on the one hand, a possible accumulation due to concentration was found [38], and on the other, in prolonged periods there was an inhibition due to the sudden decrease in photosynthesis, with the consequent arrest or slowing of maturation [39].

Water deficit also affects the phenylpropanoid, isoprenoid, and carotenoid metabolic pathways activating the expression of the transcripts correlated to glutamate and proline biosynthesis. For example, in the Chardonnay grapevine, water stress enhanced the antheraxanthin and flavonol concentrations; in the Cabernet Sauvignon, it swayed the abscisic acid metabolic pathway (9cis-epoxy carotenoid dioxygenase transcript copiousness) [40]. With high ambient light levels, the berries had the maximum Quercetin-3-glucoside levels (a harmful compound in Sangiovese grapes with a possible precipitate in the wine after the hydrolysis of glycosides with aglycon supersaturation [41]) and a lesser proportion of Malvidin-3-cumarylglucoside, compared to the shaded ones. In warm and arid climates, overhead bunch degree exposure is not helpful to excellent anthocyanin storage and synthesis [42].

Therefore, a polite approach to water resources turns out to be a component of primary importance for plant and berry development. For these reasons, zeolite soil application was investigated in a lot of research, in which the effects on land hydraulic capacities were fixed [43]. In fact, Bernardi et al. (2013) [44] indicated that joining zeolitic Brazilian sedimentary rocks to soils can sharpen their water-holding capacity (WHC). These hydrated tectoaluminosilicates of alkaline/alkaline earth elements [45] are retained as an important natural inorganic soil improver that can enhance the land’s physical/chemical properties (i.e., infiltration rate [46], cation exchange capacity [47], and saturated hydraulic conductivity [48]). It was made extensively clear that soil adaptation using zeolitic equipment refines water holding and minimizes the percolation which lowers water enforcement in agricultural management [49,50,51,52].

Considering the above, this experiment was created to improve soil management, the well-being of the vine, and the quality of the grapes through the application to the soil of a new land conditioner called “Zeowine”, derived from the compost processes of industrial wine, waste, and zeolite. The interaction of the beneficial results of Zeowine (soil + grapevines) was evidenced by the embellishment of nutritional and water efficiency, the minimizing of the need for fertilizers, the closure of the production cycle of waste material from the supply chain, and the improvement of the quality of the wines.

## 2. Results

### 2.1. Weather Parameters

**Figure 1:**
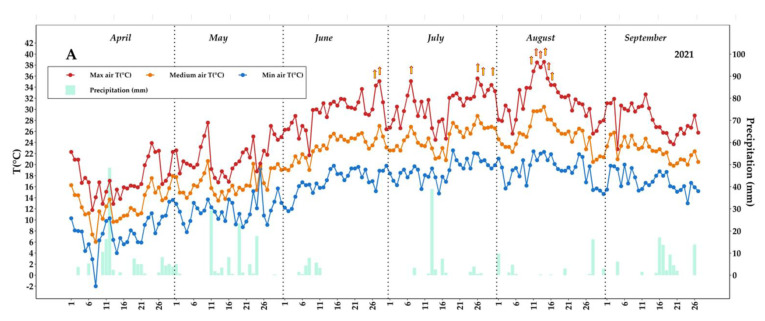

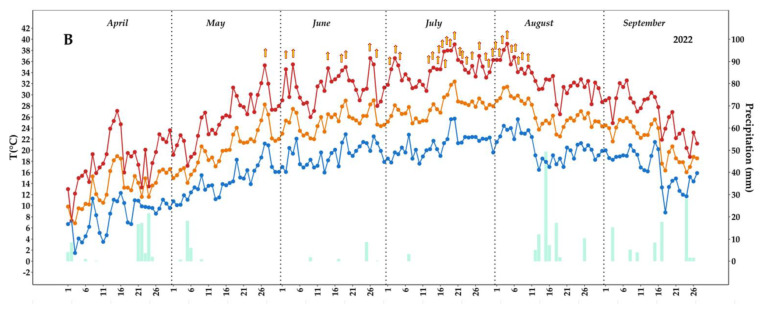
Weather patterns of the experiment location. Daily mean, maximum, and minimum air temperature (°C) and rainfall (mm) were measured from April to September (2021–2022, (**A**) and (**B**)). The arrows indicate the days during which the maximum temperature exceeded 34 °C.

Figure 1 highlights the weather patterns of the area in the 2021 and 2022 growing seasons.

Daily minimum, average, and maximum air temperatures were registered in both seasons of 2021–2022 (from April to September). The 2022 grape harvest unfolded as being more scorching and less rainy during the trial months (from April to July). The rainfall summation was as follows: 128.90 mm in April 2021, 98.10 mm in May 2021, 23.20 mm in June 2021, 61.00 mm in July 2021, 39.40 mm in August 2021, and 72.30 mm in September 2021; 74.70 mm in April 2022, 25.70 mm in May 2022, 11.60 mm in June 2022, 3.20 mm in July 2022, 103.10 mm in August 2022, and 114.00 mm in September 2022. In 2022, the rainfall was concentrated in the final phase, late August and September. The monthly averages of the max temperatures were as follows: 17.52 °C in April 2021, 21.32 °C in May 2021, 29.52 °C in June 2021, 30.73 °C in July 2021, 31.53 °C in August 2021, and 28.03 °C in September 2021; 18.31 °C in April 2022, 28.11 °C in May 2022, 31.48 °C in June 2022, 34.55 °C in July 2022, 32.79 °C in August 2022, and 25.98 °C in September 2022.

### 2.2. Ecophysiological Survey (Gaseous Exchange), Midday Stem Water Potential, and Leaf Chlorophyll a Fluorescence

**Figure 2:**
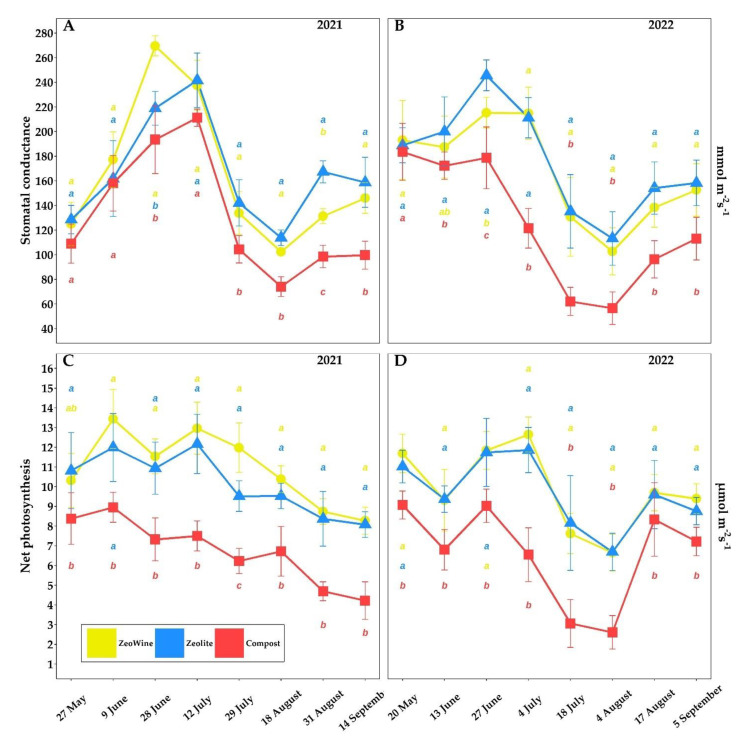
Physiological parameters (semel). Net photosynthesis (PN) and stomatal conductance (gs) of *Vitis vinifera* with three different soil management treatments. Measurements were conducted from May to September (2021 and 2022, (**A**)–(**D**)). Data (mean ± SE, *n* = 10) were subjected to one-way ANOVA. The bars represent the standard deviation. Different letters indicate significant differences between Zeowine, Zeolite, and Compost (LSD test, *p* ≤ 0.05).

**Figure 3:**
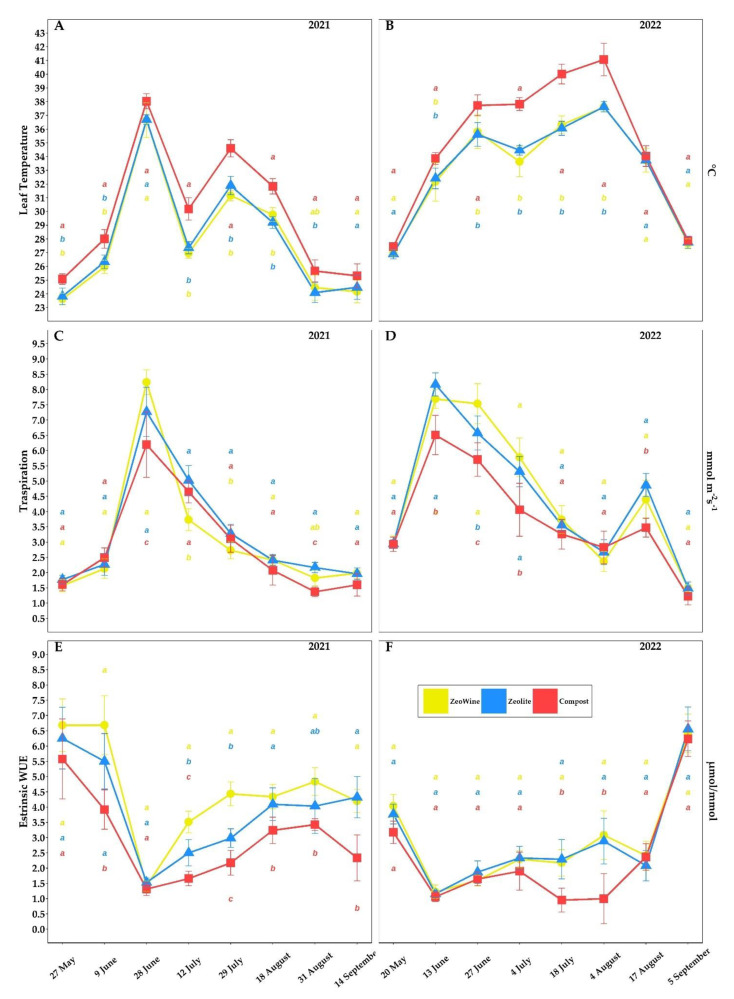
Physiological parameters (bis). Leaf temperature (°C), transpiration (E), and extrinsic water use efficiency (eWUE) of *Vitis vinifera* with three different soil management treatments. Measurements were conducted from May to September (2021 and 2022, (**A**)–(**F**)). Data (mean ± SE, *n* = 10) were subjected to one-way ANOVA. The bars represent the standard deviation. Different letters indicate significant differences between Zeowine, Zeolite, and Compost (LSD test, *p* ≤ 0.05).

**Figure 4:**
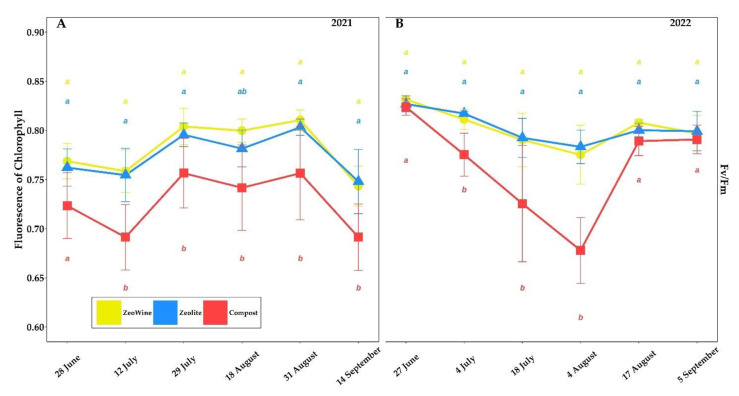
Physiological parameters (ter). Fluorescence of chlorophyll (Fv/Fm) of *Vitis vinifera* with three different soil management treatments. Measurements were conducted from June to September (2021 and 2022, (**A**,**B**)). Data (mean ± SE, *n* = 10) were subjected to one-way ANOVA. The bars represent the standard deviation. Different letters indicate significant differences between Zeowine, Zeolite, and Compost (LSD test, *p* ≤ 0.05).

**Figure 5:**
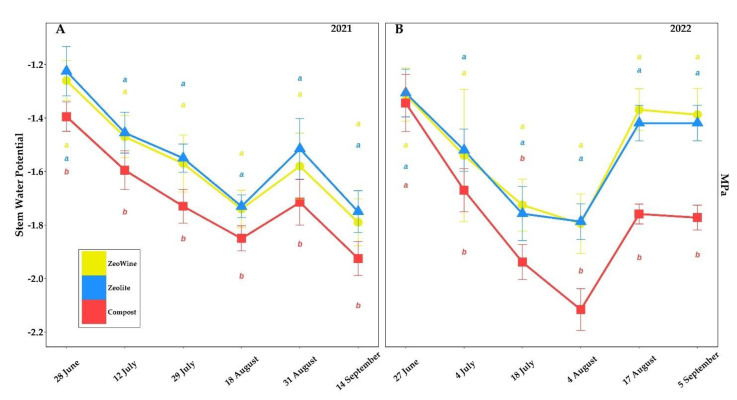
Physiological parameters (quater). Stem water potential (Ψstem) of *Vitis vinifera* with three different soil management treatments. Measurements were conducted from June to September ((**A**) 2021 and (**B**) 2022). Data (mean ± SE, *n* = 10) were subjected to one-way ANOVA. The bars represent the standard deviation. Different letters indicate significant differences between Zeowine, Zeolite, and Compost (LSD test, *p* ≤ 0.05).

The *Vitis vinifera* ecophysiological parameters according to three different land treatments (Zeowine, zeolite, and compost) are indicated in Figure 2, Figure 3, Figure 4 and Figure 5.

Stomatal conductance and net photosynthesis follow the seasonal trend. Significant differences in net photosynthesis and stomatal conductance during the seasons were found. Generally, no differences were ever found between the Zeowine and the zeolite treatments. Observing the 2022 vintage in stomatal conductance, differences emerge as early as June. In both years, the compost recorded lower values in net photosynthesis in each measure of the season (2021 and 2022).

The transpiration rates reflect the trend of temperatures and rainfall during the two years. Particularly in the hottest moments, significant differences in leaf temperatures, eWUE, and transpiration during the seasons were found. During the less torrid vintage, there were almost never differences between the treatments in the transpiration. Significantly higher leaf temperatures were found in the compost treatment (from June to August 2021 and 2022).

In the Zeowine and zeolite grapevines in both vintages, higher values of Fv/Fm were customarily found. No differences were recorded in June 2021 and 2022 and in September 2022.

Significant discrepancies in the water potential parameters (Ψstem) in the 2021–2022 seasons were registered. The compost treatment from July showed clearly more negative values of water potential. The 2022 vintage was classified as the driest and most torrid of the two years, reaching water potentials of less than −2.0 MPa (August 2022). During 2021, the following decrements were found in the compost compared to the Zeowine and the zeolite, respectively: 10.71% and 13.87% (28 June), 8.50% and 9.62% (12 July), 10.19% and 11.61% (29 July), 6.32% and 6.93% (18 August), 8.54% and 13.20% (31 August), and 7.54% and 10.00% (14 September). During 2022, the following decrements were found in the compost compared to the Zeowine and the zeolite, respectively: 2.38% and 2.87% (27 June), 8.44% and 9.86% (4 July), 12.37% and 10.31% (18 July), 17.88% and 18.39% (4 August), 28.49% and 23.96% (17 August), and 27.77% and 24.89% (5 September).

### 2.3. Berry Quality

**Figure 6:**
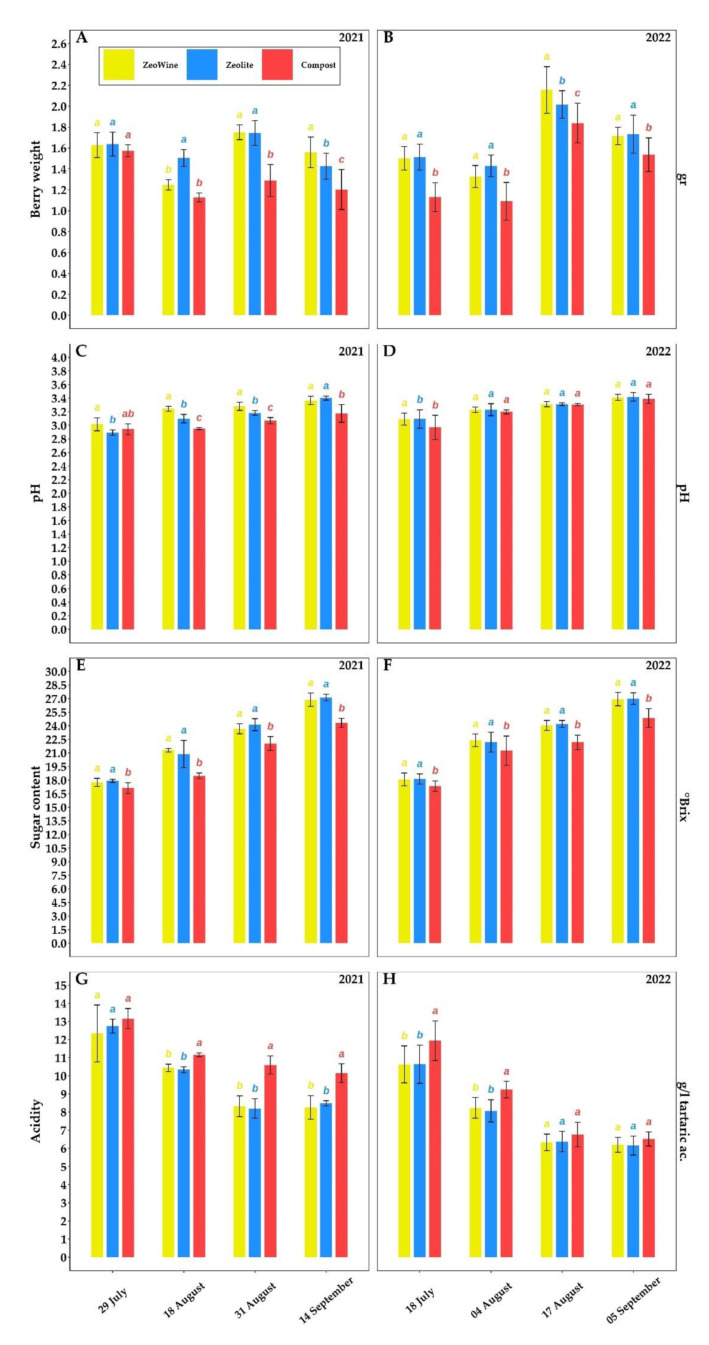
Technological maturity. Sugar content (°Brix), total acidity (TA), pH, and berry weight of *Vitis vinifera* treated with Zeowine, Zeolite, and Compost during two seasons (2021–2022, (**A**)–(**H**)). Measurements were conducted four times: full veraison (29 July 2021 and 18 July 2022), mid-maturation (18 August 2021 and 4 August 2022), full maturation (31 August 2021 and 17 August 2022), and harvest (14 September 2021 and 5 September 2022). Data (mean ± SE, *n* = 10) were subjected to one-way ANOVA. The bars represent the standard deviation. Different letters indicate significant differences between Zeowine, Zeolite, and Compost (LSD test, *p* ≤ 0.05).

**Figure 7:**
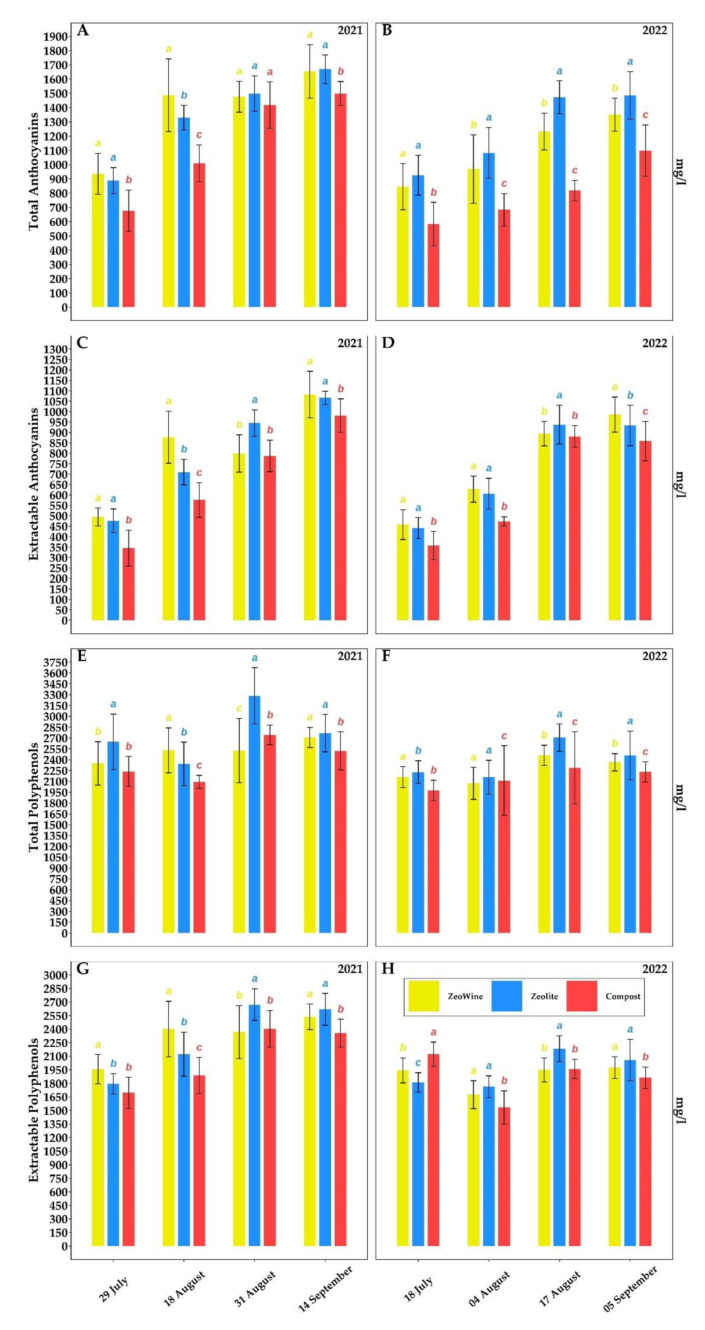
Phenolic maturity. Total and extractable anthocyanins, total and extractable anthocyanins of *Vitis vinifera* treated with Zeowine, Zeolite, and Compost during two seasons (2021–2022, (**A**)–(**H**)). Measurements were conducted four times: full veraison (29 July 2021 and 18 July 2022) and (18 August 2021 and 4 August 2022), full maturation (31 August 2021 and 17 August 2022), and harvest (14 September 2021 and 5 September 2022). Data (mean ± SE, *n* = 10) were subjected to one-way ANOVA. The bars represent the standard deviation. Different letters indicate significant differences between Zeowine, zeolite, and compost (LSD test, *p* ≤ 0.05).

**Figure 8:**
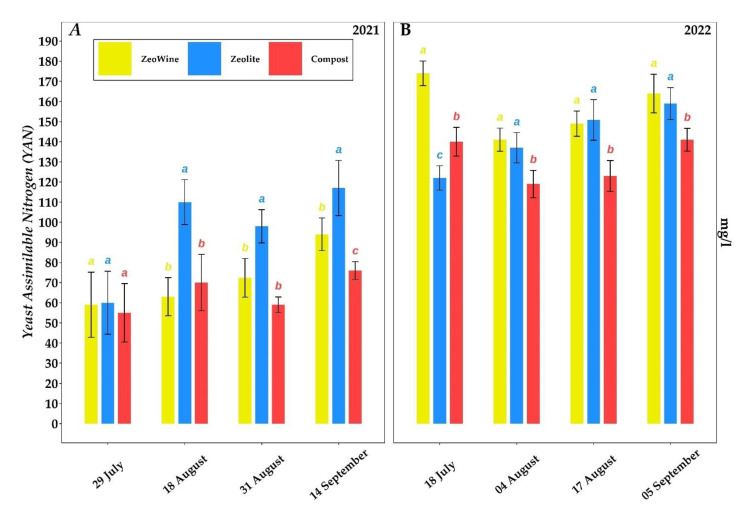
Yeast assimilable nitrogen (YAN). Yeast assimilable nitrogen (YAN) of *Vitis vinifera* treated with Zeowine, zeolite, and compost during two seasons (2021–2022, (**A**) and (**B**)). Measurements were conducted four times: full veraison (29 July 2021 and 18 July 2022), mid-maturation (18 August 2021 and 4 August 2022), full maturation (31 August 2021 and 17 August 2022), and harvest (14 September 2021 and 5 September 2022). Data (mean ± SE, *n* = 10) were subjected to one-way ANOVA. The bars represent the standard deviation. Different letters indicate significant differences between Zeowine, Zeolite, and Compost (LSD test, *p* ≤ 0.05).

Figure 6, Figure 7 and Figure 8 and Table 1 and Table 2 expose, over the two years, the typesetting of the berries of *Vitis vinifera*: technological maturity and phenolic maturity.

Basically, no difference was found between the Zeowine and the zeolite. Instead, differences were seen between the compost and the other two treatments. The compost treatment proved to be the one characterized by a smaller berry, lower sugar content, and higher acidic content. Regarding the weight of the berry, the following increases in Zeowine and zeolite were found compared to the compost treatment on the harvest date: 29.69% and 18.62% (14 September 2021) and 11.70% and 12.89% (5 September 2022). While in the sugar content, the following increases in Zeowine and zeolite were found compared to the compost treatment on the harvest date: 10.52% and 11.51% (14 September 2021) and 8.32% and 8.60% (5 September 2022).

A difference was found between the compost and the Zeowine/zeolite treatments. The treatments with clinoptilolite added proved to be the two characterized by a higher anthocyanin and polyphenol content (both total and extractable). In the extractable anthocyanin content, during 2021, the following increments were found in the Zeowine and zeolite treatments, respectively, as compared to the compost one: 43.30% and 37.93% (29 July), 52.31% and 23.21% (18 August), 1.46% and 19.99% (31 August), and 10.27% and 8.67% (14 September). While during 2022, the following were found: 28.22% and 23.43% (18 July), 32.75% and 28.13% (4 August), 1.58% and 6.47% (17 August), and 14.86% and 8.65% (5 September).

During harvest, the yeast assimilable nitrogen content proved to be significantly higher in the zeolite treatment in 2021 (117 mg/L) and in the Zeowine treatment in 2022 (164 mg/L).

The study shows that the anthocyanin profile of Sangiovese grapevines is characterized by the prevalence of Malvidin-3-glucoside over the other di-oxygenated and tri-oxygenated anthocyanins. In fact, the sum of the trisubstituted anthocyanins (Delphinidol-3-glucoside, Malvidol-3-acetylglucoside, Malvidol-3-cumarylglucoside, Petunidin-3-glucoside, and Malvidol-3-glucoside) was higher than that of the disubstituted ones (Cyanidol-3-glucoside, Peonidol-3-acetylglucoside, Peonidol-3-cumarylglucoside, and Peonidol-3-glucoside). The disubstituted anthocyanins were as follows: 3 August 2021: 25.1 ZW, 29.5 Z, 31.1 C; 17 August 2021: 33.6 ZW, 35.0 Z, 26.4 C; 3 September 2021: 30.8 ZW, 14.7 Z, 35.8 C; and 14 September 2021: 28.8 ZW, 21.9 Z, 31.9 C. The trisubstituted anthocyanins were as follows: 3 August 2021: 74.9 ZW, 70.4 Z, 68.9 C; 17 August 2021: 66.4 ZW, 64.9 Z, 73.7 C; 3 September 2021: 69.3 ZW, 85 Z, 64.2 C; and 14 September 2021: 71.3 ZW, 78.1 Z, 68.2 C. Significant differences joined to the treatments in Cyanidin-3-glucoside, Malvidin-3-acetylglucoside, Malvidin-3-cumarylglucoside, Malvidin-3-glucoside, Peonidin-3-glucoside, and Petunidin-3-glucoside were recorded; whereas, conversely, their amount was not interesting in the phenological stage. The malvidin + peonidin + petunidin (methoxylated anthocyanins) to cyanidin + delphinidin (non-methoxylated anthocyanins) ratios were as follows: 3 August 2021: 2.76 ZW, 2.10 Z, 2.12 C; 17 August 2021: 1.72 ZW, 1.89 Z, 2.15 C; 3 September 2021: 2.04 ZW, 7.01 Z, 1.95 C; and 14 September 2021: 2.53 ZW, 3.33 Z, 1.97 C. No hydroxycinnamic acids derivatives were observed. In fact, in the 2021 vintage, no ferulic, caffeic, or coumaric acid content was found in the grapes. Instead, the flavonol derivatives of quercetin (glucoside, rutinoside, glucuronide, and galactoside) and kaempferol (glucoside) were identified. Here, the derivatives of quercetin were the most depicted. No myricetin derivatives were detected. The compost treatment showed a greater accumulation of quercetin during ripening and at harvest (Quercetin-3-O-glucoside, Quercetin-3-O-galactoside, and Quercetin-3-O-glucuronide); furthermore, it showed a higher content of Kaempferol-3-Oglucoside.

The 2022 vintage confirmed the characterization of the anthocyanin profile of Sangiovese and its division into anthocyanidins. No difference was found in the fractionation percentages. The disubstituted anthocyanins were as follows: 3 August 2021: 27.9 ZW, 29.3 Z, 15.9 C; 17 August 2021: 25.0 ZW, 32.3 Z, 29.5 C; 3 September 2021: 24.1 ZW, 22.7 Z, 26.8 C; and 14 September 2021: 29.9 ZW, 31.2 Z, 33.4 C. The trisubstituted anthocyanins were as follows: 3 August 2021: 72.0 ZW, 70.8 Z, 84.2 C; 17 August 2021: 74.9 ZW, 67.7 Z, 70.5 C; 3 September 2021: 76.0 ZW, 77.4 Z, 73.2 C; and 14 September 2021: 69.9 ZW, 68.8 Z, 66.6 C. Significant differences joined to the treatments in Cyanidin-3-glucoside, Malvidin-3-acetylglucoside, Malvidin-3-cumarylglucoside, and Peonidin-3-glucoside were recorded; whereas, conversely, their amount was not interesting in the phenological stage. The malvidin + peonidin + petunidin (methoxylated anthocyanins) to cyanidin + delphinidin (non-methoxylated anthocyanins) ratios were as follows: 3 August 2021: 2.11 ZW, 2.38 Z, 4.21 C; 17 August 2021: 2.18 ZW, 2.41 Z, 2.22 C; 3 September 2021: 3.03 ZW, 3.06 Z, 2.54 C; and 14 September 2021: 2.68 ZW, 2.35 Z, 2.32 C. Traces of ferulic, coumaric, and caffeic acids were monitored. The compost treatment showed a greater accumulation of quercetin during ripening and at harvest (Quercetin-3-O-glucoside, Quercetin-3-O-galactoside, and Quercetin-3-O-glucuronide). Irrespective of the treatment, in 2022 the amount of quercetin was more abundant with respect to 2021.

### 2.4. Principal Component Analysis

**Figure 9:**
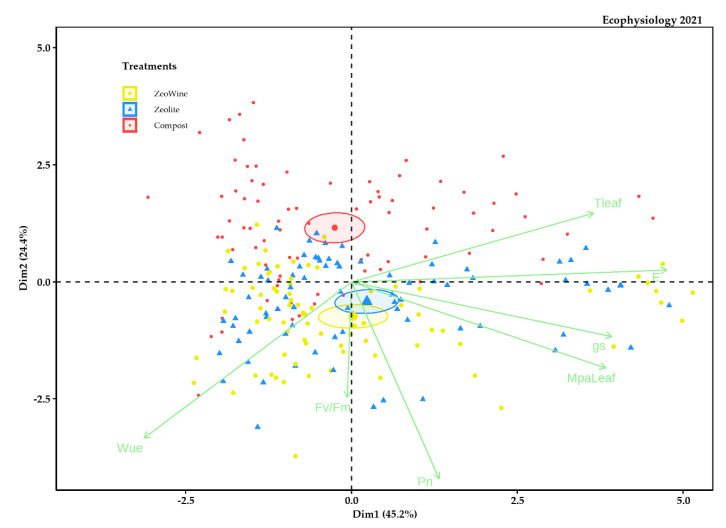
PCA ecophysiology 2021 season. PCA of the following variables (27 May, 9 June, 28 June, 12 July, 29 July, 18 August, 31 August, and 14 September): stem midday water potential, net photosynthesis, transpiration, leaf temperature, stomatal conductance, the fluorescence of chlorophyll, and water use efficiency.

**Figure 10:**
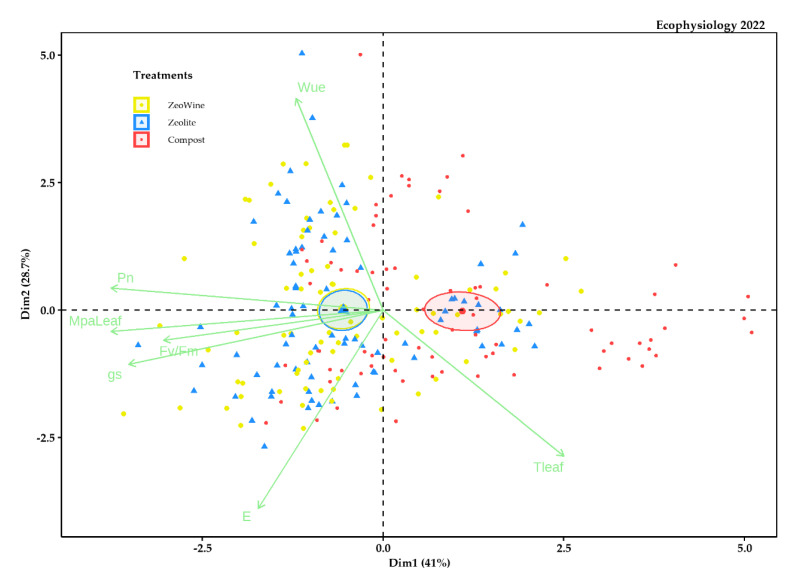
PCA ecophysiology 2022 season. PCA of the following variables (20 May, 13 June, 27 June, 4 July, 18 July, 4 August, 17 August, and 5 September): stem midday water potential, net photosynthesis, transpiration, leaf temperature, stomatal conductance, the fluorescence of chlorophyll, and water use efficiency.

**Figure 11:**
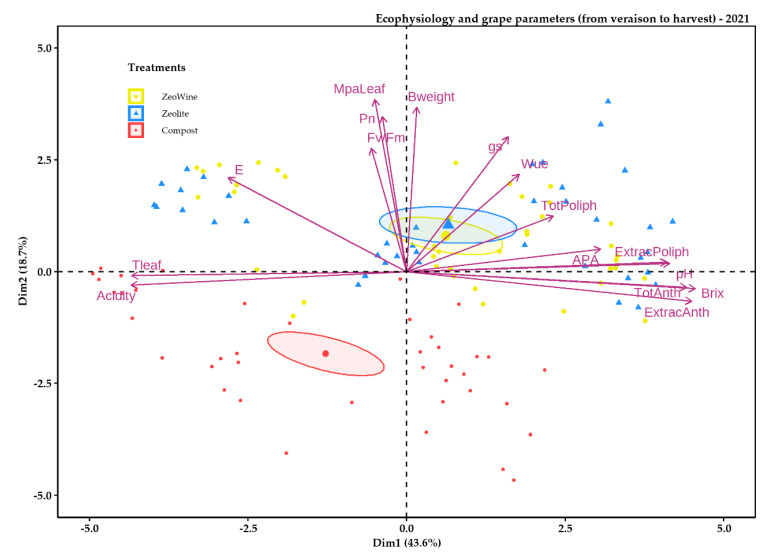
PCA ecophysiology and grape parameters 2021 season. PCA of the following variables (29 July, 18 August, 31 August, and 14 September): stem midday water potential, net photosynthesis, transpiration, leaf temperature, stomatal conductance, the fluorescence of chlorophyll, water use efficiency, sugar content, pH, acidity, total and extractable polyphenol, total and extractable anthocyanins, and YAN.

**Figure 12:**
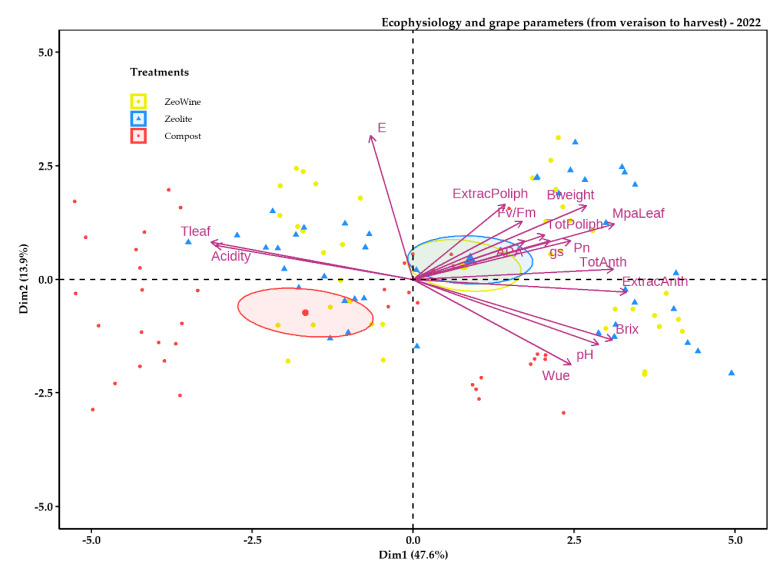
PCA ecophysiology and grape parameters 2022 season. PCA of the following variables (18 July, 4 August, 17 August, and 5 September): stem midday water potential, net photosynthesis, transpiration, leaf temperature, stomatal conductance, the fluorescence of chlorophyll, water use efficiency, sugar content, pH, acidity, total and extractable polyphenol, total and extractable anthocyanins, and YAN.

The PCA analyses were examined in order to synthetize all the details in an individual elucidatory graph. The PCA described almost 40% of the variability of the data (Figure 9, Figure 10, Figure 11 and Figure 12). As is illustrated, the PCA bracketed the variables into three specific clusters, depending on their bearing during the season.

The compost treatment was to the upper part of the distribution and positively related to the transpiration and leaf temperature and negatively related to eWUE and Fv/Fm (Dim1 45.2%). Instead, PC 2 (Dim2) explained 24.4% of the data variability.

The Zeowine and zeolite treatments were to the left part of the distribution and negatively related to PN and eWUE and positively related to TLeaf (Dim1 41.0%). Instead, PC 2 (Dim2) explained 28.7% of the data variability.

The compost treatment was to the down part of the distribution and positively related to the phenolic parameters and negatively related to acidity, Fv/Fm, E, and water potential (Dim1 43.6%). Instead, PC 2 (Dim2) explained 18.7% of the data variability.

The Zeowine and zeolite treatments were to the right part of the distribution and negatively related to E, acidity, and TLeaf and positively related to the phenolic parameters (Dim1 47.6%). Instead, PC 2 (Dim2) explained 13.9% of the data variability.

### 2.5. Production

The production of the treatments was measured at the harvest stage (14 September 2021 and 5 September 2022, Figure 13).

In both seasons (2021 and 2022), no difference in the number of bunches was monitored. The Zeowine and zeolite treatments differed significantly from the compost one by the following factors: total yield per grapevine and bunch weight. The lower values of these two parameters were noted in the compost one.

## 3. Discussion

Global warming and inaccurate agricultural habits are the main factors biassing berries and wine esteem in Mediterranean viniculture [53]. These factors can provoke a soluble solid discharge, together with a decline in anthocyanin content, acidity, and productivity [54]. The aftermath produces slacking (or stuck) fermentations and economic shrinkage in the winery [55]. Furthermore, insensitivity and non-respect for the vineyard ecosystem conservation induced by agronomic choices not aimed at recycling or revaluing the product lead to environmental pollution (the use of synthetic products) [56] on one hand and on the other to greater waste production (the non-closed loop approach) [57,58]. This experimentation was created to improve vine welfare and berry quality through the Zeowine application, a new amendment derived from the compost processes of industrial wine waste and zeolite.

In our study, it was confirmed that environmental agents, such as temperature, soil moisture, and light radiation affect water potential [59,60]. In the water potential parameter, in both seasons, significant differences were recorded between the compost and the two treatments with zeolite owing to the clinoptilolite property (i.e., augmented H_2_O retention capacity [61]). The compost treatment during the driest times recorded the following negative percentage decreases compared to the other two (Zeowine and zeolite): vintage 2021, −10.19% and −11.61% on 29 July, −6.32% and −6.94% on 18 August; vintage 2022, −12.39% and −10.31% on 18 July, 17.88% and −18.39% on 4 August. In fact, we suppose that thanks to the zeolitic ability to retain and release water [62] (up to 60% of its weight) in a reversible way without changing its microporous and crystalline structure [63], the treatments with clinoptilolite alleviated the unfavorable results of water stress thanks to the better management of rainwater and water reserves by increasing the availability of water for the vines [64] in drought conditions [65].

From the point of view of gas exchanges, constant monitoring from May to September highlighted significant differences, especially between the compost treatment and the other two, respectively. The stomatal conductance in the days where the maximum temperatures reached critical values underwent a significant decrease in the compost, recording the following stress values: 74.10 mmol m^−2^s^−1^ on 18 August 2021, 98.53 mmol m^−2^s^−1^ on 31 August 2021, 62.10 mmol m^−2^s^−1^ on 18 July 2022, 56.60 mmolm^−2^s^−1^ on 4 August 2022, and 96.30 mmolm^−2^s^−1^ on 17 August 2022. This stomatal regulation is most probably primed by the abscisic acid in the leaves (ABA), in partnership with other quicker hydraulic signals (cavitations or embolisms) [66]; these markers happen in the xylem vessels when the atmospheric request cannot be satisfied by the water content of the vineyard soil. This generates a tightness inside the tracheid or xylem vessel and an excess of gas molecules from the water (i.e., hydraulic conductivity dwindling) [67]. Moreover, we hypothesized an association with advanced VPD (vapor pressure deficit) that leads to reduced carbon assimilation (lower stomatal conductance) [68] without necessarily reducing the transpiration (E) rate to the same measure. On the other hand, Scholasch et al. (2009) [69] highlighted that for a given water supply rank, elevated VPD rates tend to enhance grapevine transpiration when solar radiation is continuous. In Spain, with arid regimes, Balbontín (2015) [70] reported morning minimum (0.5–1.5 kPa data range) and maximum noon data (4.5–6.0 kPa).

Overall, the photosynthesis rates were lesser in the non-treated plants compared with the Zeowine and zeolite grapevines. The desirable rates of PN per unit/leaf/area for basal health in uncovered vine leaves fluctuate from 6 to 18 μmol m^−2^ s^−1^ [71,72]. During the elevated temperature spell, the PN in the compost leaves declined remarkably; it was shown that as a consequence of the high radiation a 30–50% drop in PN can occur, with markedly declining rates during the occurrence of heatwaves [73,74]. Although there was also a physiological declension in photosynthesis in the Zeowine and zeolite plants, this flexure did not affect the performance of the electron transport chain. In fact, the monitored values did not reach stress thresholds, probably thanks to the help of the zeolite [75] being able to soak up the carbon dioxide molecules [76], increasing the total CO_2_ adjacent to the stomata. Furthermore, we surmise that this improvement was also related to the mitigating outcome of the zeolite at high leaf temperatures [77], which would negatively influence the trek of the carbohydrates from the leaf by affecting their photosynthetic activity (feedback down-regulation) [78].

Transpiration during the hottest periods also showed differences; Zeowine and zeolite were the treatments with the highest transpiration rate. Even though a full mechanistic comprehension of the transpiration rate under elevated temperature stress status is missing, the literature states that such a rejoinder involves different biophysical or physiological processes [79], such as a modification in membrane permeability [80], a rise in cuticle permeability [81], and a lower water viscosity [82]. As demonstrated by Naveed et al. (2020) [83], in their work developed to assess the occurrences of an endophytic bacterium (*Caulobacter* sp.) added to the zeolite on *Sesamum indicum* L., the gaseous exchange values (e.g., transpiration) and water connections were tightened by the co-application of compost and zeolite.

A further limitation was monitored in the compost treatment. The significant abatement in the Fv/Fm (chlorophyll fluorescence parameter) resulted in an increase in energy dissipation in the antenna complex with the probable degradation of the D1 protein [84] (reduction in photosystem II efficiency, i.e., photoinhibition) [85].

As indicated in several works, technological maturity was swayed by water stress (a significant difference in midday water potential) [86] and by temperature stress (a significant difference in leaf temperature) [87]. The results found by Wang et al. (2003) [88] showed that high water deficiency obstructs sugar unloading in the berry. Additionally, the discharging of sugar phloem during the maturation is through the apoplastic system, and this scheme demands energy input [89]. In accordance with these explanations, the compost treatment showed a more delayed ripening than the other two: a lower sugar content, an acidic content unsuitable for a grape harvest (10.14 g/L during the 2021 harvest against the canonical 5–8 g/L) (Frost et al., 2017), and undeveloped berry weight. The disposability of water influences the sugar concentration of the berries in a different and complex way since, on the one hand, a greater availability leads to a major concentration of sugar due to a greater PN activity [90] and, on the other hand, it can lead to a lower concentration by dilution with the berry growth [91]. After an alteration of the water supply, even in the most recent genomic and transcriptomic approach (deep RNA sequencing approach; [92]), when sampling is performed on the same date [93], as in our trial, gene expression modifications were reported. Our results are confirmed in the test realized by Santesteban and Royo (2006) [94], where in order to reach a correct maturation it is necessary to have ratios between the leaf area and the production of at least 5–10 cm^2^/g up to 15–17 cm^2^/g to allow the correct photosynthesis.

The plants that had clinoptilolite applications showed a greater weight of the berry, confirming the beneficial effect of these tectoaluminosilicates on production [95,96]. Probably in addition to the better management of the water resource, the zeolites increased the substrate cation exchange capacity [97], allowing a better and gradual granting of nutrients [98,99] and avoiding losses due to leaching [100]. The effect on the weight of the berries involves cell division or/and cell expansion modifications [101].

The yeast assimilable nitrogen amount denotes changes according to the year; only during 2022, the values reached congruous thresholds to avoid the additions of inorganic nitrogen (diammonium phosphate DAP) or inorganic ammonia added to the primary amino nitrogen (AMM + PAN) [102] (> 140 mgNL^− 1^; necessary for efficient fermentation) [103]. The zeolite intake improved the YAN concentrations in both growing seasons. This result is attributable to the zeolitic ability to exchange cations such as the ammonium cation [104] (NH4+), one of the main parameters soaked up by the plants’ plasma membrane [105].

As suggested by González-Sanjosé and Diez (1992) [106], berry skin sugars show a role as regulators in anthocyanin synthesis and, generally, of phenols. We found that the treatments (Zeowine and zeolite) with greater sugar accumulation in parallel recorded a greater content of polyphenols and anthocyanins (both total and extractable). In general, comparing the two vintages, during 2022 we measured lower absolute values compared to the less torrid vintage. In fact, the temperature (high and low) during maturation, particularly during the 3° stage, presumably conditioned the abscisic acid degradation and production in the berry skins; the endogenous abscisic acid levels sway the VvmybA1 gene expression that drives anthocyanin biosynthetic expression [107]. In addition, high nocturnal temperatures can quell the gene expression of dihydroflavonol 4-reductase, leucoanthocyanidin dioxygenase, chalcone synthase, flavanone 3-hydroxylase, and flavonoid 3-O-glucosyltransferase, causing minor expression levels of anthocyanin biosynthetic genes during the beginning of ripening [108]. Moreover, another factor in addition to anthocyanin degradation could be represented by the mRNA transcription inhibition of the anthocyanin biosynthetic genes [109].

This study shows that the anthocyanin profile of (SG) Sangiovese grapevines is typified by the preponderance of Malvidin-3-glucoside [110] over the other di-oxygenated and tri-oxygenated anthocyanins. In fact, the sum of the trisubstituted anthocyanins (Delphinidol-3-glucoside, Malvidol-3-acetylglucoside, Malvidol-3-cumarylglucoside, Petunidin-3-glucoside, and Malvidol-3-glucoside) was higher than that of the disubstituted ones (Cyanidol-3-glucoside, Peonidol-3-acetylglucoside, Peonidol-3-cumarylglucoside, and Peonidol-3-glucoside). In addition, the acylated pigments in the Sangiovese berries are scarce [111]. Contrary to what de Rosas et al. (2022) [112] pointed out, the treatments did not affect either the percentage of anthocyanins or the acylated forms. In our study, we cannot suggest acylation as an eventual stress-response gear for reducing the unfavorable incidents caused by high temperature.

The 2022 severe climatic context may have caused a superior ratio of methoxylated/non-methoxylated anthocyanins in berry skins with respect to 2021 (17 August and 3 September 2022). In fact, high temperature and solar radiation precipitate the changeover from the hydroxylated (delphinidin and cyanidin) [113] to the methoxylated derivatives of anthocyanins (malvidin, petunidin, and peonidin) [114]. The methoxylation activity depicts a metabolic process that affects the stability of the different anthocyanins, giving them minor susceptibility to non-enzymatic or enzymatic oxidation under tricky and stressful regimes [115], stabilizing the phenolic B ring and causing a red shift in the absorption spectrum [116]. The treatments, generally, did not sway the ratio between methoxylated and non-methoxylated. Contrary to what Tarara et al. (2008) [117] demonstrated, the absolute concentrations of the dihydroxylated anthocyanins (cyanidin and peonidin; red anthocyanins) and the trihydroxylated (delphinidin, malvidin, and petunidin; purple and blue anthocyanins) [118] did not undergo substantial changes in either the treatment or the vintage effect.

Among the non-flavonoid polyphenols, gallic acid (a hydroxybenzoic acid—GA; 3_4_5-trihydroxy benzoic acid) [119], which is chiefly stored as galloylated flavan-3-ols [120], showed an increment during the 2022 season for all treatments. In fact, its content is biased by preharvest environmental status [121]. Contrary to what Del Castillo Alonso et al. (2020) [122] found, we saw that hydroxybenzoic acids were probably susceptible to temperature variations. Additionally, in agreement with Xi et al. (2010) [123], their content was enhanced by improving land management habits (at harvests, ZW and Z showed superior content). Therefore, these applications could increase the co-pigmentation between GA and malvidin-3-Oglucoside in red wine (stabilizing role) [124].

Four glycosylated forms of quercetin (flavonols class) (glucosides, galactosides, rutinosides, and glucuronides) as 3-O-glycosylated were found [125]. In both years, significantly higher doses of quercetin in the compost treatment were found. We suppose that this high quantity was correlated to their biosynthesis being influenced by temperature stress and sunlight exposure; in fact, the concentration was found to be 4–8 times less in the shaded cluster [126] (photo-protector role). However, considering the recent studies on this compound in Sangiovese grapes [127,128], this increase was found to be depleted in the finished product. Sangiovese wine can produce a quercetin precipitate during its aging from the glycosides hydrolysis (i.e., supersaturation of the aglycons) [41]. Conversely, the grapes that underwent clinoptilolite applications recorded lower quercetin contents.

Many authors showed that the absorption and checked relief of moisture by zeolite ameliorated the growth and plant yield under drought stress conditions [129,130,131]. In conformity with these results, in our experiment we also noticed an increase in production in the Zeowine and zeolite treatments. The greater yield of both vintages was attributed to a greater weight of the bunch and not to a different number of bunches. The clinoptilolite porous framework might have helped to keep the ground moist and ventilated [132] (less compactness and greater humidity in the periods of development of the berry). In addition, it might have retained principal nutrients (N, Mg, P, B, and K) in the root zone [133] for reuse by the vine when requested.

## 4. Materials and Methods

### 4.1. Experimental Project, Place of Setting, and Composting Process

The trial was organized at CMM (Cosimo Maria Masini Estate) (Lat 43°41′ N—Long 10°53′ E), Italy. CMM is nestled in the San Minato hills, Poggio a Pino Street (PI), in Tuscany: an antique medieval hamlet, located along the route of the historic Via Francigena. Since the end of MCMXCVIII, it has belonged to the Masini family, Tuscan entrepreneurs engaged in activities related to the environment and research in the name of sustainable development. The attention to sustainability directed the property to apply, from the very beginning, cultivation and winemaking methods without the use of chemicals.

The experiment was executed on 21-year-old organic vines (*Vitis vinifera* L., 1753) in two plant cultivation vintages (i.e., 2020 and 2021). The plants taken into consideration are of the red Sangiovese cultivar (clonal selection CCL 2000/3), on Kober 5 BB rootstock (*Vitis berlandieri* × *Vitis riparia*); they are cultivated with a vertical upward trellis and pruned as a spurred cordon.

From the analysis of the company’s soil, a clayey-calcareous soil with the presence of a rocky skeleton emerges (clay 51.9%; sand 17.4%; silt 30.7%; active limestone 170 g/kg; pH 8.1; CSC 21.5 meq/100 g; organic matter 2.1%).

Using an experimental randomized block design, ten blocks per treatment were established; every block consisted of 4 rows; 10 vines per treatment were selected for the measurements. The experiment with three treatments, the Zeolite (Z), Compost (C), and Zeowine (ZW), was set up. Zeowine is a product made by combining the properties of zeolite (clinoptilolite) with the stable organic substance of a compost obtained on a company scale from the reuse of processing waste from grapes, pomace, and stalks. CMM provided the wastes from the 2020 and 2021 harvest (grape skins, stalks, and vineyard pruning waste), which were shredded to 4–5 cm and processed for their composting. The optimal dimensions and typology of the zeolite (Zeocel Italia, PI, Italy) to be used for the production of Zeowine was selected (85% clinoptilolite) with a granulometry of 0.2–2.5 mm, which was identified in order to ensure better aeration of the heaps during composting. For the first composting cycle (start of composting, 11/11/2020) CMM proceeded to prepare three different kinds of composting heaps: the 3 heaps of about 9 tons each with zeolite and organic residues at the ratio 1:2.5 w:w of fresh weight; a heap with zeolite and organic residues at the ratio 1:10 w:w of fresh weight; and a control heap (without zeolite). The two additional kinds of composting heaps were prepared with about 2 tons of waste to demonstrate the efficiency of the presence of zeolite at different rates in improving the composting system and the quality of the end product during the whole experimentation, with respect to the control heap without zeolite.

**Figure 14:**
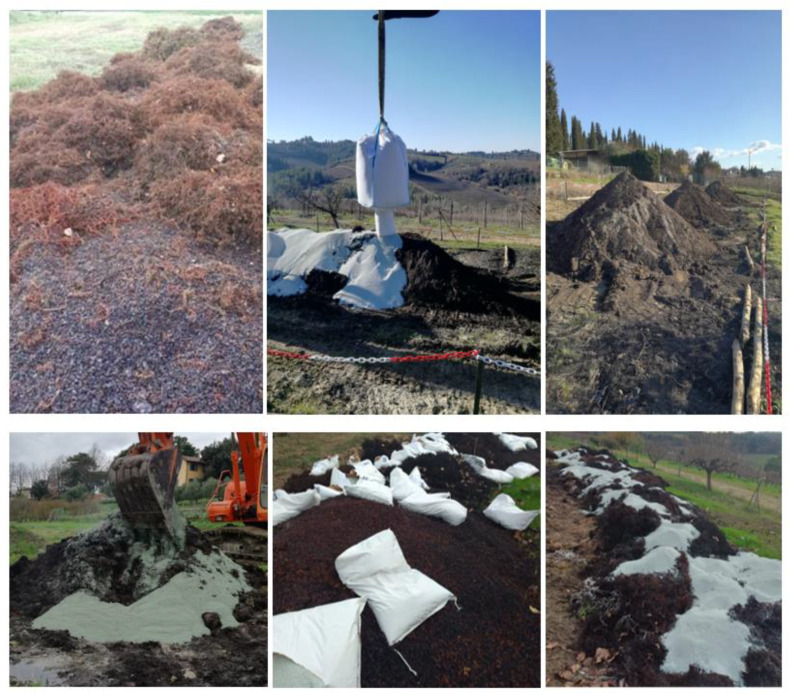
Composting cycle at CMM.

On the basis of the results obtained from the first composting cycle at CMM, in the second composting cycle (start of composting, 12/12/2021), the following piles were prepared at CMM: n. 2 piles of about 9 tons each with zeolite and vine wastes at the ratio 1:10 *w/w*; n. 1 piles of about 9 tons each with zeolite and vine wastes at the ratio 1:2.5 *w/w*; n. 1 control piles of about 9 tons with 100% vine wastes (Figure 14).

Briefly, to facilitate the aerobic compost-making success, mechanical turnings were performed every 30 days for the 150 days of composting with periodical irrigations until the moisture content was >40% [134,135].

The temperature of all the heaps rapidly increased from the beginning of the experimentation. In the control heap, the thermophilic phase (temperature higher than 55 °C) was reached after two weeks, while in the heaps with zeolite it was recorded after three–four weeks. A temperature greater than 55 °C during this stage is extremely important to kill the pathogens, thus achieving the sanitization of the raw material [136,137]. The maximum temperature was measured in the control heap after 18 days (65 °C), while in the heaps with zeolite it was reached after about 34–38 days from the beginning of composting (60–63 °C). The thermophilic phase was maintained for 12 days in the control heap (days 16 to 28), for 24 days in the heap with 1:2.5 zeolite:compost (days 24 to 48), and for 32 days in the heap with 1:10 zeolite:compost (days 22 to 54).

Similar results were also reported by Himanen and Hänninen (2009) [138], who claimed that the duration of the thermophilic stage increased from 2 to 3 weeks following the addition of commercial elements (i.e., zeolite, ashes, kaolinite, chalk, and sulfates) to a biowastes + peat mixture.

In the control heap, the temperature decreased and reached the mesophilic stage (temperature lower than 50 °C) after 30 days from the beginning of the composting process. However, this stage was reached after 52 and 64 days in the heaps with zeolite 1:2.5 and 1:10, respectively. During the mesophilic stage, the zeolite heaps showed a higher temperature with respect to the control heap. In fact, Venglovsky et al. 2005 [139] demonstrated that the presence of zeolite during the composting system, enhancing the porosity of the compost, can enable better aeration for metabolic heat generation by aerobic microorganisms with respect to the control heap. At the end of the thermophilic period, from the turning operations, it was possible to observe the actual state of maturation of the material in which neither the grape stalks nor the pomace were still recognizable, and the assumed consistency was that of mature compost. The complete maturation of Zeowine was achieved after roughly 150 days of composting (Table 3).

**Figure 15:**
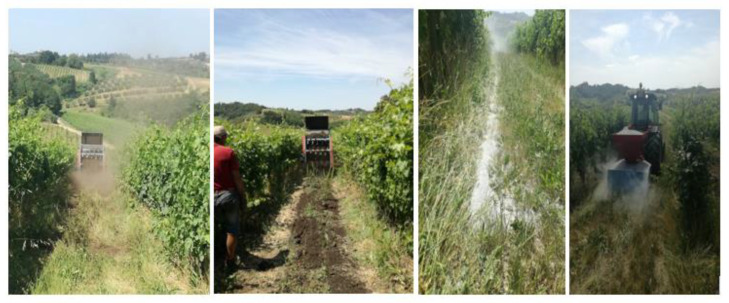
Treatment applications at CMM.

**Figure 16:**
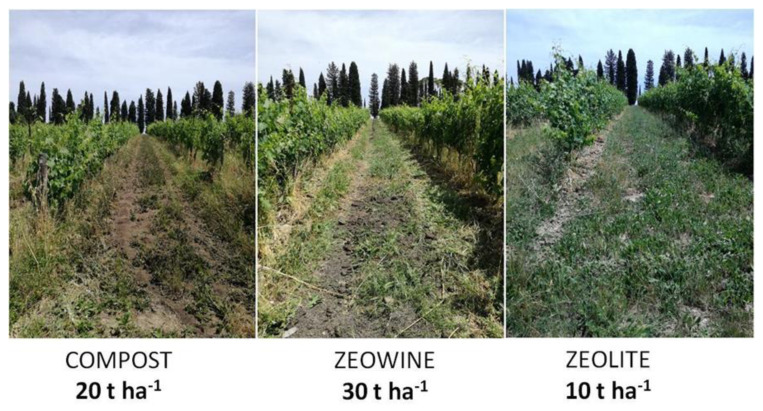
Treatment application results at CMM.

The application of treatments was executed on 1.2 ha of vineyard in production with a spreader manure (Figure 15 and Figure 16) in the spring: Zeowine 30 t/ha, zeolite 10 t/ha, and compost 20 t/ha [140]. A surface tillage (15–20 cm) for the burial of the treatments was carried out. After the date reported in Table 3, 1:2.5 treatments were selected for the experiment. In fact, the objective of the preliminary implemented experiments was to define the best zeolite:compost ratio to be used in the experiment and to scrupulously follow the composting process.

The agro-meteorological system Pre-meteo (Mybatec S.R.L., NO, Italy) monitored the main parameters such as rainfall (mm) and air temperatures (°C).

### 4.2. Ecophysiological Survey (Gaseous Exchange), Midday Stem Water Potential, and Leaf Chlorophyll a Fluorescence

Ecophysiological surveys (between 10:55 a.m. and 12:55 p.m.) were conducted on the tagged vines (10 replicates per treatment) every week, from May to the harvest: in 2021, 27 May, 9–28 June, 12–29 July, 18–31 August, and 14 September; in 2022, 20 May, 13–27 June, 4–18 July, 4–17 August, and 4 September. The following parameters were accounted for with the following method, °C (leaf temperature), gs (stomatal conductance), PN (photosynthesis), and E (transpiration), adopting Ciras 3PP Systems, USA (390–400 ppm CO_2_, surrounding temperature IR Thermometry, RGBW Control Red 38%, Green 37%, Blue 25%, White 0%, automatic zero/diff bal mode, and 1300 μmol m^−2^s^−1^ photon flux) [141]. eWUE (extrinsic water use efficiency) was estimated from the PN/E ratio [142].

On the same leaves between 12:45 and 13:45 p.m., the stem midday water potential (Ψstem) was valued by a Scholander pressure chamber (600-type, PMS Instrument Co, Albany, OR, USA) [143]. The surveys were conducted on the tagged vines (10 replicates per treatment) every week, from June to the harvest (the beginning of the summer period with higher temperatures): in 2021, 28 June, 12–29 July, 18–31 August, and 14 September; in 2022, on 27 June, 4–18 July, 4–17 August, and 4 September.

On the same days, chlorophyll fluorescence (Fv/Fm) was gauged with a fluorometer (Handy-PEA^®^, Hansatech Instruments, Norfolk, UK), adapting leaves in the dark for 30 min, following the Maxwell and Johnson calibration [144].

### 4.3. Berry Quality

In each treatment, 100 berries (per replication) were arbitrarily chosen to develop the technological maturity. Firstly, the berries of each treatment were independently weighed with the Kern PCD model (a precision digital scale). The sample was squeezed to analyze the sugar content (expressed in Brix degree), total acidity (expressed in g L-1 tartaric acid), and pH. The following tools and products were employed for technological analysis: a portable optical refractometer (RHA-503), a pH meter (HHTEC), bromothymol blue, glass burettes, and a sodium hydroxide solution (NaOH-0.1 M).

In each treatment, 100 more berries (per replication) were arbitrarily chosen to develop phenolic maturity. Total and extractable polyphenols and total and extractable anthocyanins were estimated by the Glories method [145].

The determination of nine major anthocyanins (Cyanidin-3-glucoside, Delphinidin-3-glucoside, Malvidin-3-acetylglucoside, Malvidin-3-cumarylglucoside, Malvidin-3-glucoside, Peonidin-3-acetylglucoside, Peonidin-3-cumarylglucoside, Peonidin-3-glucoside, and Petunidin-3-glucoside) in the musts was performed according to OIV MA AS315 11: R2007 1 Method OIV MA AS315 11 TypeII method HPLC-Determination, by an external laboratory (ISVEA), under the analysis conditions proposed by Resolution Oeno 22/2003, changed by Oeno 12/2007 [146]. In addition, with high-performance liquid chromatography-high resolution mass spectrometry (HPLC-HRMS) [147], Coumaric Acid, Gallic Acid, Caffeic Acid, Ferulic Acid, Kaempferol-3-O-glucoside, Quercetin-3-O-glucoside, Quercetin-3-O-rutinoside, Quercetin-3-O-galactoside, and Quercetin-3-O-glucuronide were evaluated. The berry samples were kept at −80 °C until they demanded analysis. The determination of yeast assimilable nitrogen (as the sum of amino and ammoniacal nitrogen) in musts was performed with an enzymatic colorimetric kit (Steroglass, S. Martino Campo—Pg, Italy).

Finally, the cluster number per vine, the weight of the bunch per vine, and the total yield/vine were determined at harvest with a digital scale (VAR model, Italy) (10 grapevines per treatment).

### 4.4. Statistical Analysis

The data and graphs were processed with R version 4_0_3.—RStudio (R Development Core Team) (Tidyverse packages [148]), first with the Shapiro–Wilk and Levene tests, then with one-way ANOVA (*p* ≤ 0.05). The means comparison was performed with the Tukey HSD test [149] (*p* ≤ 0.05). PCA [150] (principal component analysis) was exploited to fix the connections among specific variables under investigation and to distinguish between the different treatments [151].

## 5. Conclusions

Global warming and inaccurate agriculture can provoke soluble solid discharge, together with a decline in anthocyanin content, acidity, and productivity. Furthermore, non-respect for the vineyard ecosystem conservation induced by agronomic choices not aimed at recycling or revaluing the product leads to environmental pollution on one hand and, on the other, to greater waste production. Our results seem to argue that, in the years marked by low water disposability, severe water deficiency is a narrowing coefficient for the anthocyanin potential in Sangiovese grapes and that Zeowine or zeolite applications could preserve it. The absence of adjuvant in the soil (compost treatment) leads to a lower production (lower yield per vine), characterized by an excess of quercetin in the must and a lower color (slowed ripening). The Zeowine and zeolite treatments were the most balanced ones for the ecophysiological parameters (water potential and net photosynthesis), grape quality (sugar and anthocyanin content), and berry weight.

On the basis of the following achieved results (the demonstrated efficacy of Zeowine in improving the performance of the vineyard soils and the characteristics of the grapes) in operational practice it would be desirable to define and implement protocols for composting waste from the viticultural chain with zeolite and protocols for the application of the product on vine plants by introducing the culture of the circular economy and the valorization of waste in companies in order to promote the environmental, economic, and social sustainability of companies.

## Figures and Tables

**Figure 13:**
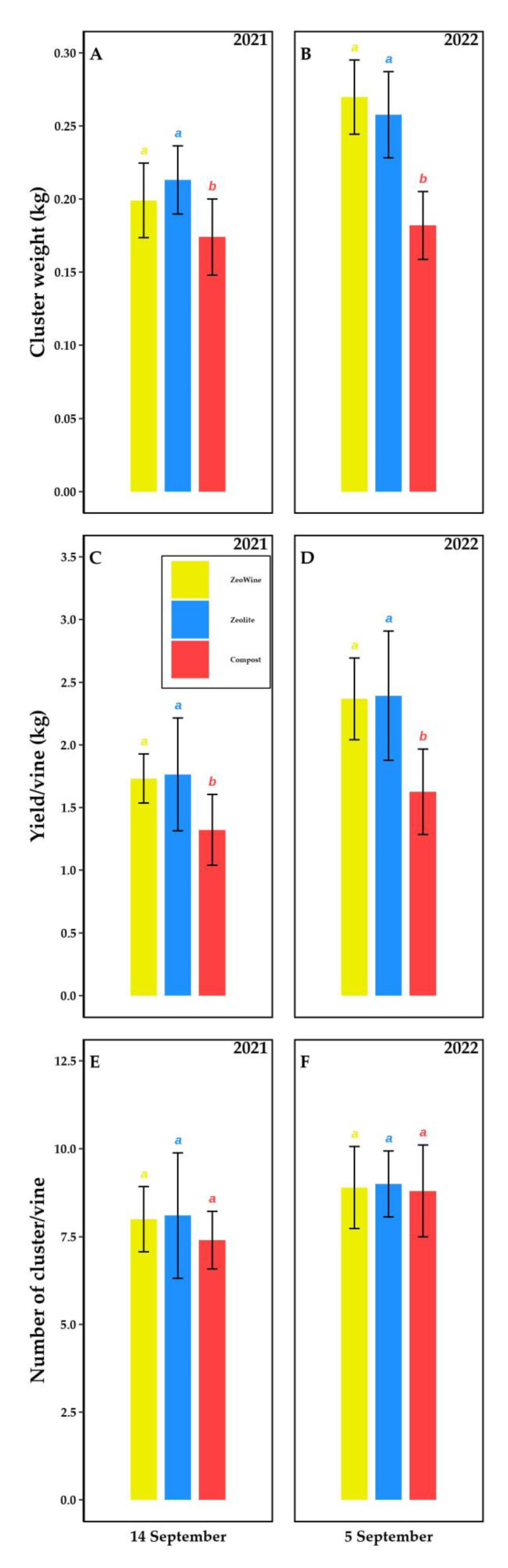
Productive parameters. Cluster weight, yield per vine, and the number of clusters per vine (2021 and 2022 seasons, (**A**)–(**F**)). Measurements were at the harvest stage (14 September 2021 and 5 September 2022). Data (mean ± SE, *n* = 10) were subjected to one-way ANOVA. The bars represent the standard deviation. Different letters indicate significant differences between Zeowine, Zeolite, and Compost (LSD test, *p* ≤ 0.05).

**Table 1:** Phenolic maturity. Fractionation of anthocyanins (Cyanidin-3-glucoside, Delphinidin-3-glucoside, Malvidin-3-acetylglucoside, Malvidin-3-cumarylglucoside, Malvidin-3-glucoside, Peonidin-3-acetylglucoside, Peonidin-3-cumarylglucoside, Peonidin-3-glucoside, and Petunidin-3-glucoside) and Coumaric Acid, Gallic Acid, Caffeic Acid, Ferulic Acid, Kaempferol-3-O-glucoside, Quercetin-3-O-glucoside, Quercetin-3-O-rutinoside, Quercetin-3-O-galactoside, and Quercetin-3-O-glucuronide of *Vitis vinifera* treated with Zeowine, Zeolite, and Compost during the 2021 season. Measurements were conducted four times: full veraison (29 July 2021 and 18 July 2022), mid-maturation (18 August 2021 and 4 August 2022), full maturation (31 August 2021 and 17 August 2022), and harvest (14 September 2021 and 5 September 2022). Data (mean ± SE, *n* = 10) were subjected to one-way ANOVA. Different letters indicate significant differences between Zeowine, Zeolite, and Compost (LSD test, *p* ≤ 0.05).

	*6 August 2021*			*18 August 2021*			*31 August 2021*			*14 September 2021*			u.m.
	Zeowine	Zeolite	Compost	Zeowine	Zeolite	Compost	Zeowine	Zeolite	Compost	Zeowine	Zeolite	Compost	
**Cyanidin-3-glucoside**	12.90 ±4.25 a	17.30 ±6.18 a	18.10 ±2.12 a	20.80 ±1.66 a	19.20 ±3.52 a	15.00 ±3.04 a	17.20 ±3.31 a	5.50 ±3.47 b	19.90 ±2.88 a	14.20 ±5.12 a	10.20 ±4.21 b	17.60 ±3.07 a	%
**Delphinidin-3-glucoside**	13.70 ±1.37 a	14.90 ±2.14 a	13.90 ±2.25 a	15.90 ±2.15 a	15.30 ±2.47 a	16.70 ±1.07 a	15.70 ±4.18 a	7.00 ±3.52 a	13.90 ±3.47 a	14.10 ±5.01 a	12.90 ±3.92 a	16.10 ±2.17 a	%
**Malvidin-3-acetylglucoside**	<0.10 ±0.00 a	1.70 ±0.62 a	<0.10 ±0.00 a	<0.10 ±0.00 a	<0.10 ±0.00 a	<0.10 ±0.00 a	<0.10 ±0.00 b	7.40 ±3.44 a	0.80 ±0.12 b	<0.10 ±0.00 a	<0.10 ±0.00 a	<0.10 ±0.00 a	%
**Malvidin-3-cumarylglucoside**	<0.10 ±0.00 a	1.20 ±0.53 a	<0.10 ±0.00 a	<0.10 ±0.00 a	<0.10 ±0.00 a	<0.10 ±0.00 a	0.40 ±0.22 b	10.60 ±2.74 a	0.90 ±0.18 b	<0.10 ±0.00 a	0.60 ±0.19 a	<0.10 ±0.00 a	%
**Malvidin-3-glucoside**	42.90 ±5.78 a	35.40 ±6.24 b	37.60 ±3.56 ab	32.50 ±4.18 a	32.30 ±4.36 a	37.90 ±4.42 a	35.30 ±4.69 b	50.50 ±4.25 a	32.50 ±4.81 b	39.70 ±4.65 b	47.30 ±4.21 a	33.50 ±4.16 b	%
**Peonidin-3-acetylglucoside**	<0.10 ±0.00 a	<0.10 ±0.00 a	<0.10 ±0.00 a	<0.10 ±0.00 a	<0.10 ±0.00 a	<0.10 ±0.00 a	<0.10 ±0.00 a	0.50 ±0.11 a	<0.10 ±0.00 a	<0.10 ±0.00 a	<0.10 ±0.00 a	<0.10 ±0.00 a	%
**Peonidin-3-cumarylglucoside**	<0.10 ±0.00 a	<0.10 ±0.00 a	<0.10 ±0.00 a	<0.10 ±0.00 a	<0.10 ±0.00 a	<0.10 ±0.00 a	<0.10 ±0.00 a	0.70 ±0.19 a	<0.10 ±0.00 a	<0.10 ±0.00 a	<0.10 ±0.00 a	<0.10 ±0.00 a	%
**Peonidin-3-glucoside**	12.20 ±1.47 a	12.20 ±1.37 a	13.00 ±1.98 a	12.80 ±2.96 a	15.80 ±3.04 a	11.40 ±2.38 a	13.60 ±2.93 ab	8.50 ±2.27 b	15.90 ±2.45 a	14.60 ±3.86 a	11.70 ±2.04 a	14.30 ±2.12 a	%
**Petunidin-3-glucoside**	18.30 ±3.67 a	17.20 ±3.23 a	17.40 ±2.37 a	18.00 ±4.23 a	17.30 ±4.04 a	19.10 ±3.34 a	17.90 ±2.50 a	9.50 ±2.13 b	16.10 ±2.32 a	17.50 ±2.45 a	17.30 ±2.61 a	18.60 ±1.44 a	%
**Caffeic Acid**	n.d. ±0.00 a	n.d. ±0.00 a	n.d. ±0.00 a	n.d. ±0.00 a	n.d. ±0.00 a	n.d. ±0.00 a	n.d. ±0.00 a	n.d. ±0.00 a	n.d. ±0.00 a	n.d. ±0.00 a	n.d. ±0.00 a	n.d. ±0.00 a	mg kg^−1^
**Coumaric Acid**	n.d. ±0.00 a	n.d. ±0.00 a	n.d. ±0.00 a	n.d. ±0.00 a	n.d. ±0.00 a	n.d. ±0.00 a	n.d. ±0.00 a	n.d. ±0.00 a	n.d. ±0.00 a	n.d. ±0.00 a	n.d. ±0.00 a	n.d. ±0.00 a	mg kg^−1^
**Ferulic Acid**	n.d. ±0.00 a	n.d. ±0.00 a	n.d. ±0.00 a	n.d. ±0.00 a	n.d. ±0.00 a	n.d. ±0.00 a	n.d. ±0.00 a	n.d. ±0.00 a	n.d. ±0.00 a	n.d. ±0.00 a	n.d. ±0.00 a	n.d. ±0.00 a	mg kg^−1^
**Gallic Acid**	3.28 ±1.04 a	3.18 ±0.65 a	1.02 ±0.85 a	1.48 ±0.87 a	2.39 ±0.93 a	1.78 ±0.67 a	1.60 ±1.23 a	2.02 ±1.55 a	1.24 ±1.23 a	1.17 ±1.27 a	1.78 ±1.89 a	0.94 ±1.03 a	mg kg^−1^
**Quercetin-3-O-glucoside**	25.47 ±4.26 b	33.70 ±5.27 b	47.17 ±5.18 a	49.77 ±4.21 a	29.85 ±4.55 b	41.93 ±6.36 a	48.71 ±12.73 a	34.53 ±14.45 b	48.31 ±15.76 a	64.05 ±16.34 b	55.25 ±17.28 b	76.96 ±14.61 a	mg kg^−1^
**Quercetin-3-O-galactoside**	5.75 ±3.56 a	8.57 ±3.78 a	12.73 ±4.56 a	9.18 ±2.46 a	5.80 ±3.68 a	8.15 ±4.76 a	8.06 ±3.45 a	5.98 ±3.23 a	9.46 ±2.87 a	13.50 ±4.23 ab	10.11 ±4.36 b	22.04 ±4.11 a	mg kg^−1^
**Quercetin-3-O-glucuronide**	72.48 ±10.32 c	89.04 ±15.95 b	115.55 ±18.32 a	66.60 ±11.87 b	48.06 ±14.77 c	90.05 ±18.39 a	60.71 ±12.32 b	74.67 ±17.14 a	67.80 ±15.94 ab	58.52 ±12.62 b	44.51 ±13.46 c	70.02 ±18.46 a	mg kg^−1^
**Quercetin-3-O-rutinoside**	2.69 ±1.23 a	3.56 ±1.83 a	6.96 ±2.27 a	2.50 ±1.22 a	1.44 ±1.63 a	2.85 ±1.85 a	1.74 ±1.24 a	0.49 ±0.12 a	3.33 ±1.38 a	1.73 ±0.92 a	0.96 ±0.21 a	2.92 ±0.99 a	mg kg^−1^
**Kaempferol-3-O-glucoside**	4.87 ±1.04 b	6.17 ±2.39 b	12.79 ±2.82 a	7.99 ±3.28 ab	2.28 ±1.92 b	10.15 ±3.58 a	6.27 ±2.47 a	6.61 ±2.86 a	5.92 ±2.16 a	7.53 ±4.34 ab	6.08 ±3.52 b	12.08 ±6.78 a	mg kg^−1^

**Table 2:** Phenolic maturity. Fractionation of anthocyanins (Cyanidin-3-glucoside, Delphinidin-3-glucoside, Malvidin-3-acetylglucoside, Malvidin-3-cumarylglucoside, Malvidin-3-glucoside, Peonidin-3-acetylglucoside, Peonidin-3-cumarylglucoside, Peonidin-3-glucoside, and Petunidin-3-glucoside) and Coumaric Acid, Gallic Acid, Caffeic Acid, Ferulic Acid, Kaempferol-3-O-glucoside, Quercetin-3-O-glucoside, Quercetin-3-O-rutinoside, Quercetin-3-O-galactoside, and Quercetin-3O-glucuronide of *Vitis vinifera* treated with Zeowine, zeolite, and compost during the 2022 season. Measurements were conducted four times: full veraison (18 July 2022), mid-maturation (4 August 2022), full maturation (17 August 2022), and harvest (5 September 2022). Data (mean ± SE, *n* = 10) were subjected to one-way ANOVA. Different letters indicate significant differences between Zeowine, Zeolite, and Compost (LSD test, *p* ≤ 0.05).

	*18 July 2022*			*4 August 2022*			*17 August 2022*			*5 September 2022*			u.m.
	Zeowine	Zeolite	Compost	Zeowine	Zeolite	Compost	Zeowine	Zeolite	Compost	Zeowine	Zeolite	Compost	
**Cyanidin-3-glucoside**	16.80 ±5.21 a	16.30 ±3.17 a	7.40 ±2.85 b	14.80 ±3.70 a	16.70 ±4.72 a	16.50 ±2.00 a	12.10 ±3.64 a	12.10 ±1.53 a	13.90 ±4.20 a	14.70 ±2.70 a	16.30 ±3.91 a	17.80 ±4.04 a	%
**Delphinidin-3-glucoside**	15.30 ±2.00 a	13.30 ±1.80 a	11.80 ±2.38 a	16.60 ±2.00 a	12.60 ±1.06 a	14.50 ±1.88 a	12.70 ±3.01 a	12.50 ±1.73 a	14.30 ±1.56 a	12.60 ±2.18 a	13.50±1.22 a	12.30±1.09 a	%
**Malvidin-3-acetylglucoside**	<0.10 ±0.00 b	<0.10 ±0.00 b	7.10 ±1.00 a	<0.10 ±0.00 a	<0.10 ±0.00 a	<0.10 ±0.00 a	<0.10 ±0.00 a	2.50 ±0.60 a	<0.10 ±0.00 a	<0.10 ±0.00 a	0.40±0.00 a	<0.10±0.00 a	%
**Malvidin-3-cumarylglucoside**	<0.10 ±0.00 b	<0.10 ±0.00 b	9.4 ±1.50 a	<0.10 ±0.00 a	<0.10 ±0.00 a	<0.10 ±0.00 a	0.9 ±0.10 a	3.4 ±0.15 a	0.6 ±0.10 a	0.70 ±0.04 a	0.60±0.01 a	0.50±0.01 a	%
**Malvidin-3-glucoside**	38.50 ±6.10 a	40.20 ±5.16 a	42.70 ±4.11 a	38.90 ±4.78 a	39.40 ±5.42 a	38.80 ±4.05 a	45.50 ±5.78 a	42.70 ±4.44 a	40.40 ±6.89 a	40.60 ±3.66 a	37.70±5.23 a	37.90±6.10 a	%
**Peonidin-3-acetylglucoside**	<0.10 ±0.00 a	<0.10 ±0.00 a	<0.10 ±0.00 a	<0.10 ±0.00 a	<0.10 ±0.00 a	<0.10 ±0.00 a	<0.10 ±0.00 a	<0.10 ±0.00 a	<0.10 ±0.00 a	<0.10 ±0.00 a	<0.10±0.00 a	<0.10±0.00 a	%
**Peonidin-3-cumarylglucoside**	<0.10 ±0.00 a	<0.10 ±0.00 a	1.40 ±0.10 a	<0.10 ±0.00 a	<0.10 ±0.00 a	<0.10 ±0.00 a	<0.10 ±0.00 a	<0.10 ±0.00 a	<0.10 ±0.00 a	0.50 ±0.05 a	0.40±0.05 a	<0.10±0.00 a	%
**Peonidin-3-glucoside**	11.10 ±1.05 ab	13.00 ±1.55 a	7.10 ±0.80 b	10.20 ±1.67 a	15.60 ±3.34 a	13.00 ±2.06 a	12.00 ±2.00 a	10.60 ±2.77 a	12.90 ±1.08 a	14.90 ±3.66 a	14.50±2.98 a	15.60±1.05 a	%
**Petunidin-3-glucoside**	18.20 ±2.11 a	17.30 ±3.28 a	13.20 ±1.44 a	19.40 ±4.55 a	15.70 ±4.20 a	17.20 ±3.05 a	16.90 ±2.11 a	16.30 ±2.06 a	17.90 ±2.99 a	16.00 ±3.36 a	16.60±3.77 a	15.90±1.50 a	%
**Caffeic Acid**	<0.05 ±0.00 a	<0.05 ±0.00 a	<0.05 ±0.00 a	<0.05 ±0.00 a	<0.05 ±0.00 a	<0.05 ±0.00 a	<0.05 ±0.00 a	<0.05 ±0.00 a	<0.05 ±0.00 a	<0.05 ±0.00 a	<0.05 ±0.00 a	<0.05 ±0.00 a	mg kg^−1^
**Coumaric Acid**	<0.05 ±0.00 a	<0.05 ±0.00 a	<0.05 ±0.00 a	<0.05 ±0.00 a	<0.05 ±0.00 a	<0.05 ±0.00 a	<0.05 ±0.00 a	<0.05 ±0.00 a	<0.05 ±0.00 a	<0.05 ±0.00 a	<0.05 ±0.00 a	<0.05 ±0.00 a	mg kg^−1^
**Ferulic Acid**	0.08 ±0.00 a	<0.05 ±0.00 a	<0.05 ±0.00 a	<0.05 ±0.00 a	<0.05 ±0.00 a	<0.05 ±0.00 a	0.06 ±0.00 a	0.08 ±0.00 a	<0.05 ±0.00 a	<0.05 ±0.00 a	<0.05 ±0.00 a	<0.05 ±0.00 a	mg kg^−1^
**Gallic Acid**	10.74 ±3.23 b	21.68 ±8.15 a	11.02 ±3.05 b	8.11 ±1.20 b	23.86 ±6.63 a	8.94 ±4.60 b	19.46 ±2.21 b	23.08 ±4.32 a	16.00 ±2.01 b	32.73 ±1.67 a	24.32 ±5.60 b	11.48 ±2.36 c	mg kg^−1^
**Quercetin-3-O-glucoside**	36.79 ±5.25 b	57.51 ±7.53 a	62.13 ±7.29 a	46.51 ±9.11 b	82.38 ±10.72 a	77.40 ±13.03 a	82.06 ±19.81 b	87.54 ±19.43 b	101.23 ±22.61 a	125.44 ±20.75 b	133.77 ±21.30 b	202.32 ±15.88 a	mg kg^−1^
**Quercetin-3-O-galattoside**	<0.05 ±0.00 a	<0.05 ±0.00 a	<0.05 ±0.00 a	<0.05 ±0.00 a	<0.05 ±0.00 a	<0.05 ±0.00 a	<0.05 ±0.00 a	<0.05 ±0.00 a	<0.05 ±0.00 a	<0.05 ±0.00 a	<0.05 ±0.00 a	<0.05 ±0.00 a	mg kg^−1^
**Quercetin-3-O-glucuronide**	79.34 ±13.25 c	131.30 ±18.31 b	166.59 ±15.71 a	60.18 ±15.17 b	115.61 ±19.70 a	110.77 ±21.31 a	77.67 ±16.13 b	85.53 ±20.11 b	129.22 ±25.42 a	91.41 ±22.72 b	102.17±23.16 b	147.48 ±29.42 a	mg kg^−1^
**Quercetin-3-O-rutinoside**	4.73 ±0.85 b	9.28 ±1.17 ab	11.62 ±2.73 a	2.58 ±0.35 b	13.32 ±1.26 a	12.65 ±2.00 a	2.32 ±0.38 b	2.97 ±0.38 b	9.56 ±2.51 a	1.48 ±0.33 c	7.56 ±1.37 b	13.53 ±2.90 a	mg kg^−1^
**Kaempferol-3-O-glucoside**	22.63 ±1.81 a	13.86 ±2.30 b	11.64 ±1.42 b	12.67 ±1.36 b	18.56 ±1.72 a	20.21 ±2.20 a	18.62 ±2.95 b	27.57 ±3.23 a	15.60 ±1.04 b	28.39 ±3.02 b	42.93 ±3.44 a	21.85 ±1.92 b	mg kg^−1^

**Table 3:** Zeowine traits. The analyses were carried out by the CNR-IRET—National Research Council-Research Institute on Terrestrial Ecosystems (PI), Italy.

		Zeowine 1:2.5	Zeowine 1:10	Control	D. Lgs. N° 75/2010 Green Compost
**pH**		8.26	7.95	7.37	6–8.8
**EC**	dS m^−1^	0.22	0.35	1.51	
**CSC**	C mol c kg^−1^	45.9	43.8	36.4	
**TOC**	C %	25.68	29.41	27.01	≥ 20
**TN**	TN %	1,48	1.55	1.28	
**N-NO_3_**	mg kg^−1^	73	118	196	
**N-NH_4_**	mg kg^−1^	611	540	469	
**C/N**		17.35	18.98	21.1	≤ 50
**Humic carbon**	C %	3.5	3.6	3.2	≥ 2.5
**TK**	%	1.19	0.738	0.559	
**TP**	%	0.144	0.172	0.116	
**Available K**	mg K kg^−1^	317	531	453	
**Available P**	mg P kg^−1^	328	370	568	
**Cu**	mg Cu kg^−1^	44	70	78	< 230
**Zn**	mg Zn kg^−1^	35	45	49	< 500
**Cd**	mg Cd kg^−1^	< 0.1	< 0.1	< 0.1	< 1.5
**Ni**	mg Ni kg^−1^	13	23	27	< 100
**Pb**	mg Pb kg^−1^	7.9	8.84	8.26	< 140
**Cr**	mg Cr kg^−1^	21	33	46	< 100
**Germination Index**	%	142	126	72	> 60%
**Salmonella**	CFU g^−1^	absent	absent	absent	absent
**Escherichia Coli**	CFU g^−1^	100	100	100	≤ 1000

## Data Availability

The data presented in this study are available on request from the corresponding author.
